# Coupling Single Giant Nanocrystal Quantum Dots to the Fundamental Mode of Patch Nanoantennas through Fringe Field

**DOI:** 10.1038/srep14313

**Published:** 2015-09-23

**Authors:** Feng Wang, Niladri S. Karan, Hue Minh Nguyen, Yagnaseni Ghosh, Jennifer A. Hollingsworth, Han Htoon

**Affiliations:** 1 Center for Integrated Nanotechnologies, Materials Physics and Applications Division, Los Alamos National Laboratory, Los Alamos, New Mexico 87545.

## Abstract

Through single dot spectroscopy and numerical simulation studies, we demonstrate that the fundamental mode of gold patch nanoantennas have fringe-field resonance capable of enhancing the nano-emitters coupled around the edge of the patch antenna. This fringe-field coupling is used to enhance the radiative rates of core/thick-shell nanocrystal quantum dots (g-NQDs) that cannot be embedded into the ultra-thin dielectric gap of patch nanoantennas due to their large sizes. We attain 14 and 3 times enhancements in single exciton radiative decay rate and bi-exciton emission efficiencies of g-NQDs respectively, with no detectable metal quenching. Our numerical studies confirmed our experimental results and further reveal that patch nanoantennas can provide strong emission enhancement for dipoles lying not only in radial direction of the circular patches but also in the direction normal to the antennas surface. This provides a distinct advantage over the parallel gap-bar antennas that can provide enhancement only for the dipoles oriented across the gap.

Plasmonic nanoantennas that facilitate strong electromagnetic resonances in ultra-small mode volumes^1^ not only can enhance light-matter interactions^2^,^3^,^4^,^5^ but can also tailor the emission or scattering patterns of quantum emitters coupled to them^6^,^7^,^8^,^9^ . Therefore, plasmonic nanoantennas are critical to many applications ranging from the manipulation of fluorescence^10^,^11^,^12^, light harvesting^13^ to sensing^14^. All these applications, however, necessitate the ability to precisely position or align the quantum emitters with respect to the antennas so that strong coupling between them can be induced. In the past few years, several bottom-up self-assembly approaches^15^,^16^ as well as top-down electron beam lithography-based approaches^11^,^17^ have been developed to realize the desired alignments. Notably, single nanocrystal quantum dots (NQDs) have been incorporated into 14–30 nm gaps of dimer nanoantennas by using a two-step e-beam lithography method^11^. However, for the laterally defined dimer nanoantennas, it remains a challenge to control the gap-widths with a sub-10 nm precision. In addition, the two-step e-beam lithography requires a lithography system possessing sub-10 nm alignment precision for reliable large-scale applications.

To overcome this issue, vertically aligned patch nanoantennas, in which an ultra-thin dielectric layer is sandwiched between a metallic nano-patch and a metal layer, have been recently proposed^18^,^19^. In this case, highly precise thin film deposition technologies such as atomic layer deposition (ALD) and self-assembly growth can be utilized to create ultra-thin dielectric layer necessary for achieving strong plasmonic field. Due to ultra-strong electromagnetic resonances quantum emitters embedded in this ultra-thin gap can experience a high of 3 × 10^4^ fluorescence enhancement^18^.

While this patch nanoantenna structure works very well for enhancing the fluorescence of single quantum dots (QDs) and molecules, it encounters new challenges when integrated with single core/thick-shell NQDs (i.e., giant NQDs [g-NQDs]) or g-NQDs-clusters since their sizes can be much larger than 10 nm, resulting in an uneven dielectric film thickness. Even if the QDs-clusters could be incorporated into the ultra-thin dielectric gap of patch nanoantennas, the enclosed antenna structures can still bring difficulties in detecting the cluster configuration.

As a step toward resolving this issue, we present a new coupling approach, in which g-NQDs are coupled to the fringe field of the fundamental mode of the patch antennas. (i.e. near field that leaks out of the resonator into the dielectric medium surrounding the conductor^20^). In this coupling approach, instead of inserting g-NQDs into the dielectric gap during the fabrication, they are simply placed along the edge of the antenna after the completion of all fabrication steps. A reasonably strong g-NQD-antenna coupling can be achieved by adjusting patch sizes and dielectric layer thicknesses so that the eigen-wavelength of fundamental mode would be in resonance with the emission wavelength of g-NQDs.

## Results

**Figure 1:**
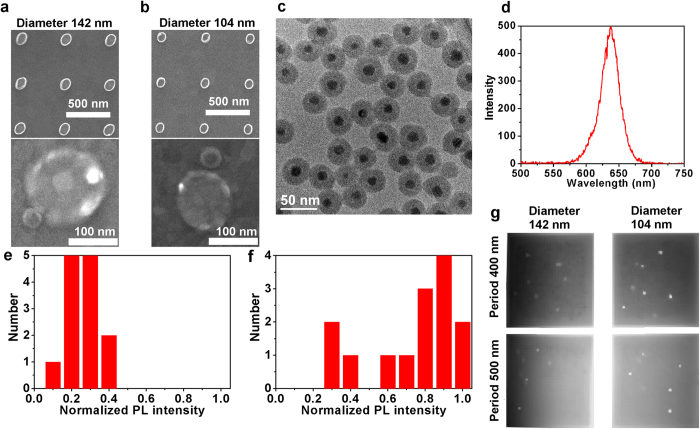
Comparison between g-NQDs coupled to 142 nm and 104 nm patch nanoantennas. (**a**,**b**) show the SEM pictures of the fabricated patch nanoantennas arrays and the formed g-NQDs-antennas coupled structures. (**a**,**b**) are for the patches of 142-nm and 104-nm diameter respectively; (**c**) TEM image and (**d**) emission spectrum of the synthesized CdSe/CdS/SiO_2_ g-NQDs; (**d**) PL intensity distribution for g-NQDs coupled to 142-nm patch nanoantennas; (**e**) PL intensity distribution for g-NQDs coupled to 104-nm patch nanoantennas; (**g**) Fluorescence image of silica coated CdSe/CdS g-NQDs on patch nanoantennas arrays with 400/500-nm periods and 142/104-nm patches.

To demonstrate this principle, we fabricated four antenna arrays of two different circular patches with 104- and 142-nm diameters and 400- and 500-nm periodicities (Fig. 1a,b). The fabrication process involves sputtering of 120 nm thick Au layer onto a cleaned fused silica substrate followed by spin coating of a ~200 nm thick PMMA (950 A4) film to define the lateral dimensions of the patches by e-beam lithography. After resist development and a slight plasma etching, 30 nm thick SiO_2_ and Au were then consecutively deposited by electron-beam evaporation and sputtering respectively. A standard lift-off process is finally applied to obtain patch antenna arrray. The silica coated non-blinking g-NQDs are then deposited onto the antenna. These g-NQDs have a 4-nm CdSe core overcoated with a 16-monolayer CdS shell and a 10–12-nm-thick SiO_2_ shell in sequence^21^,^22^,^23^,^24^ (Fig. 1c and Supporting Information S1). The measured emission spectra of the g-NQDs show a peak at 640 nm with ~30 nm spectral widths (Fig. 1d). Here, the SiO_2_ shell is employed to prevent metal- induced PL quenching of g-NQDs so that an enhancement on radiative rate can be investigated cleanly. The g-NQDs in solution were drop-casted onto the fabricated nanoantennas as well as onto a cleaned glass substrate for reference measurements. To determine whether the investigated g-NQDs are effectively coupled to the antennas, all the emission spots within the array area were optically characterized first and then were correlated with their exact SEM images. We use 22 × 22 μm array itself as a big position marker for all the QDs. The concentration of QDs solution is adjusted to form a sparse distribution of single dots with roughly 1 QD per 3 μm × 3 μm area. Then, under optical microscope, we estimate the rows and columns of the array where the g-NQDs are located. We then use the relative positions between the QDs to further make sure that the optically characterized QDs would be precisely correlated with their SEM pictures. Scanning electron microscope (SEM) investigation shows that most of g-NQDs are dispersed as single dots while a small portion form small clusters composed of 2–4 g-NQDs. In addition, a high percentage of g-NQDs (roughly 45%) were deposited within (50 nm) of the nano-patches as shown in Fig. 1a,b, providing a large probability for forming coupled structures. We speculate that the capillary force that develops during the drying of solvent around the patch antenna pulls the g-NQDs toward the patch. The emission spots which were actually small clusters and/or placed more than 30 nm away from the patches edges were excluded from analysis.

To study the emission intensity enhancement of circular patch nanoantennas with different patch diameters and periodicities, a continuous wave laser at 405 nm wavelength was used to simultaneously excite all the fabricated patch nanoantennas. The histogram (Fig. 1e,f) showing PL intensity distribution of the QDs deposited on 104- and 142-nm patch antennas revealed that the enhancement induced by the 104-nm antenna is 3–4 times stronger than by the 142 nm antenna. PL images for the antenna with different periodicities (top and bottom rows of Fig. 1g) show no significant differences indicating that periodicity have little effect on PL emission of g-NQDs. On the other hand, PL images for the patches of 104 nm diameter (right column) clearly show much stronger g-NQD emission than those for the patches of 142 nm (left column) providing the evidence that the 104-nm patch antennas enhance the PL of g-NQDs significantly.

**Figure 2:**
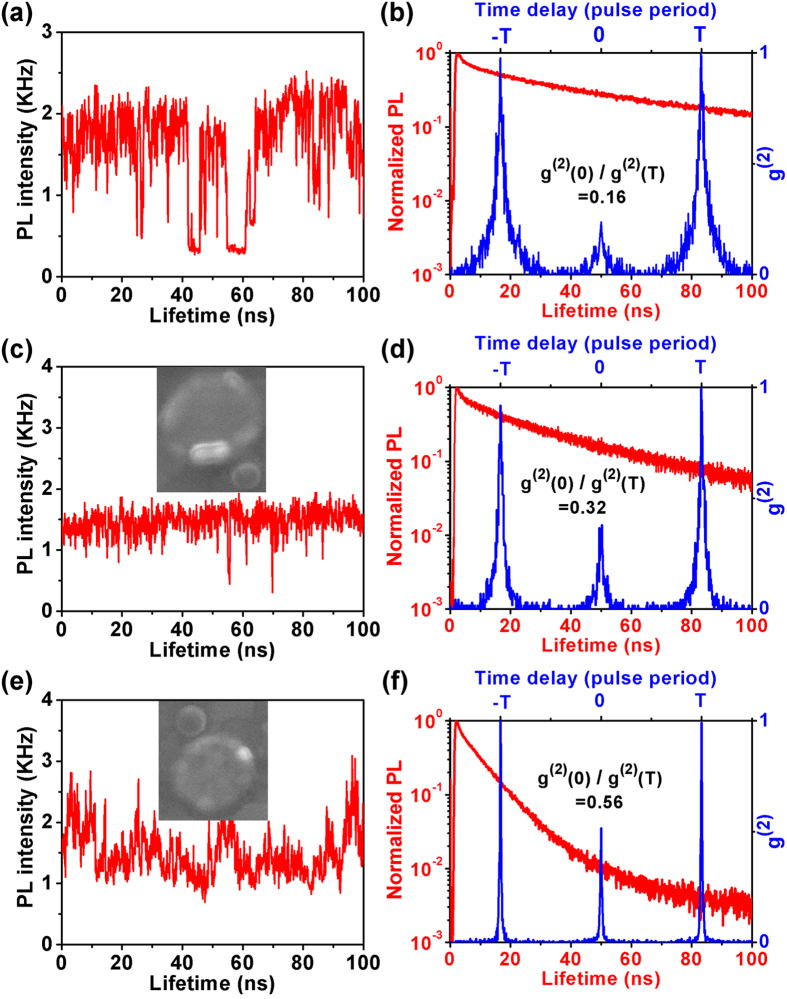
PL time traces (**a**,**c**,**e**), PL decay curves (**b**,**d**,**f**: red trace), and second order photon correlation functions (**b**,**d**,**f**: blue trace) of a referenced g-NQD (**a**,**b**), a g-NQD coupled to 142-nm patch antenna (**c**,**d**), and a g-NQD coupled to 104-nm patch antenna (**e**,**f**), respectively.

To gain further insight into this effect we performed the time-correlated single photon counting experiment for 10 (and 6) single g-NQDs coupled to the 104-nm (and 142-nm) antennas. Giant NQDs are excited with a 405 nm ps pulse laser at 1 MHz repetition rates. The power of the laser excitation is maintained to excite <0.2 excitons per pulse excitatioin. PL intensity-time traces, PL decay curves, and 2^nd^ order photon correlation functions providing a measure on relative emission efficiency of bi-exciton emission process^24^,^25^,^26^,^27^ are compared with those of referenced g-NQDs spread on the glass substrates. They are also compared with those measured on g-NQDs placed in the center of parallel gap-bar antennas utilizing two-step e-beam lithography (see Supporting Information S2)^17^,^24^. The gap-bar antennas are also designed to be in resonance with the emission band of the g-NQDs. Figure 2 shows the representative PL time traces, PL decay curves and g^(2)^ functions acquired from one referenced g-NQD on glass and 2 g-NQDs coupled to 142- and 104-nm patch antennas. PL time traces (Fig. 2a,c,e) show that average PL intensity and non-blinking behavior remains the same for all three g-NQDs. Because our g-NQDs were excited by laser pulses with excitation periods much longer than their decay times, the emission is only defined by quantum yields (QYs) and the excitation rates of the g-NQDs. The emission intensity enhancement that results from the enhancement of radiative decay rates under CW excitation cannot be observed in this pulsed excitation condition. However, the lack of any changes in average PL intensity for the g-NQDs coupled to antennas provides evidence that coupling to the antenna does not introduce any quenching that would reduce QY, nor does it provide enhancement to the excitation rate. These are expected because the SiO_2_ shell can prevent metal quenching and the antennas are not in resonance with the 405 nm excitation laser. The negligible enhancement at 405 nm wavelength is confirmed by our simulation (see Supporting Information S3) and the pump-power dependent PL intensity fitted by a Poissonian distribution model (see Supporting Information S4).

PL decay curves (Fig. 2b,d,f) on the other hand provide clear evidence on enhancement of g-NQD radiative decay. Specifically, they show that PL lifetimes decrease from ~70 ns of reference g-NQDs to 40 ns and 12 ns of g-NQDs coupled to 142- and 104-nm antennas respectively. The second order photon correlation functions (Fig. 2b,d,f) also reveal that the relative bi-exction emission efficiency (i.e., Q_2X_/Q_1X_) given by the center to side peak area ratio (Q_2X_/Q_1X_ = g^(2)^(0)/ g^(2)^(T))^25^ increase from 0.16 of a g-NQDs on glass to 0.32 and 0.56 for g-NQDs coupled to 142- and 104-nm antennas, respectively. As the radiative decay rate of the bi-exciton (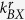) is related to that of the single exciton 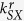 through a statistical scaling relation, 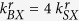, bi-exciton decay of the g-NQD coupled to the antenna is also expected to enhance at the same rate. Because the plasmonic field has no effect on the non-radiative Auger recombination rate of bi-exciton, the enhancement of radiative rate leads to the enhancement of the bi-exciton emission efficiency.

**Figure 3:**
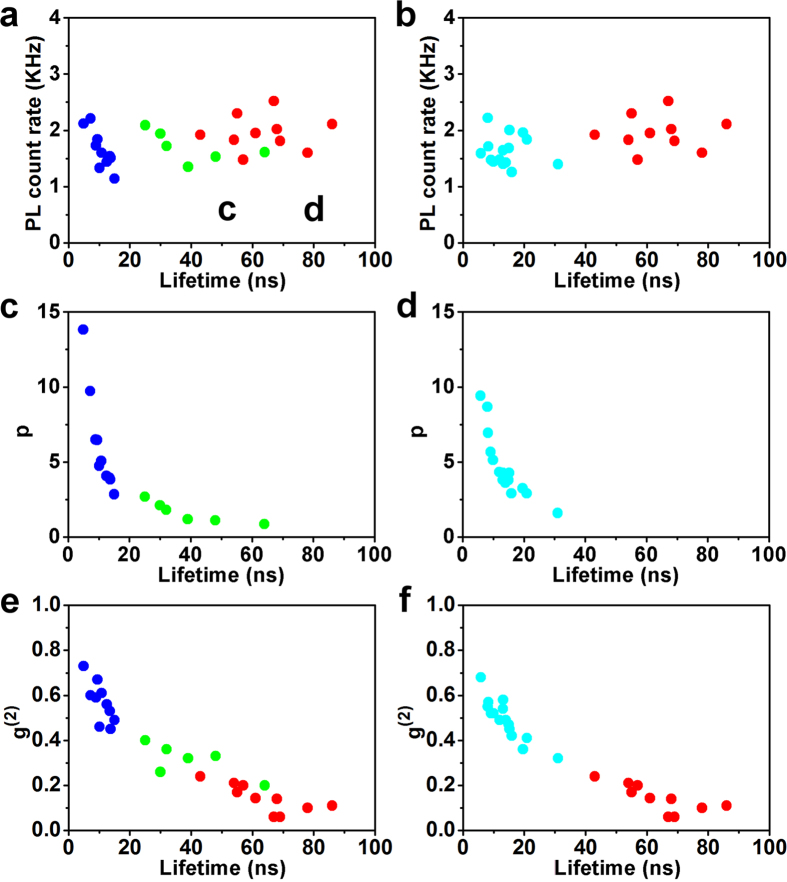
Statistical data of PL intensity, radiative rate enhancement and g^(2)^. (**a**) average PL intensity, (**c**) radiative rate enhancement factor P and (**e**) g^(2)^ as the function of lifetime for g-NQDs placed on patch nanoantennas; (**b**) average PL intensity, (**d**) radiative rate enhancement factor P and (**f**) g^(2)^ as the function of lifetime for g-NQDs placed in gap-bar nanoantennas. In (**a**,c,**e**) the blue and green circles represent the data points for 104- and 142-nm antennas, respectively. In all the figures, red circles are for the reference g-NQDs on glass.

To make a quantitative comparison, we plot average PL count rates, decay enhancement factors and g^(2)^s of reference g-NQDs, g-NQDs coupled to 142- and 104-nm patch antennas and g-NQDs placed between gap-bar antennas as the function of their PL lifetimes (Fig. 3). Further evidence on complete suppression of metal induced PL quenching is provided by the fact that PL count rates of all the g-NQDs, regardless of their coupling, are distributed in a 1.1- to 2.5-KHz range. With respect to the PL decay rate, the reference g-NQDs show the lifetime (*τ*_*ref*_) distributed in a wide range between 40 ns and 90 ns, with an average of 64 ns. At the same time, g-NQDs coupled to 104-nm patch antennas exhibit remarkably shortened lifetimes (*τ*_*antenna*_) varied within a narrow range of 4–16 ns comparable to that observed in g-NQD placed in the gap-bar antenna 5–30 ns. Lifetimes of g-NQDs coupled to 142-nm antennas on the other hand distribute in the range of 25 to 70 ns. Based on the average decay rate of reference g-NQDs (64 ns), the calculated decay rate enhancement factor P for all the dots coupled to different antenna structures are plotted in Fig. 3c,d. The plots show that P as high as 14 can be achieved with the 104-nm antenna. This value is even slightly higher than the 9.2 that is obtained on gap-bar antennas^17^ and confirms that the fringe coupling method is actually as good as that of the well-aligned gap-bar antennas.

Q_2X_/Q_1X_ values extracted from the center to side peak area ratios are plotted as the function of PL lifetimes, as in Fig. 3e. The plots show that Q_2X_/Q_1X_ values that are distributed in the 0.03–0.25 range with the average of 0.16 for reference g-NQD increased to 0.19–0.4 with an average of 0.33 for the 142-nm patch antennas and to 0.4–0.73 with an average of 0.56 for the 104-nm patch antennas, respectively. This enhancement of Q_2X_/Q_1X_ is also comparable to those observed for g-NQDs placed in gap-bar antennas, as shown in Fig. 3f. As explained above, we can understand this enhancement as a direct consequence of the plasmonic enhancement of the radiative recombination process.

## Discussion

**Figure 4:**
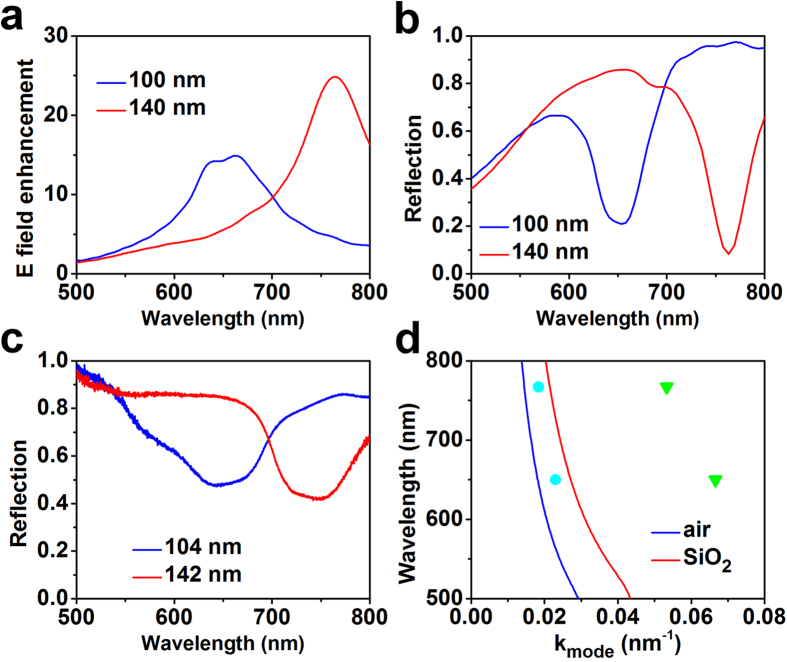
Spectra and dispersion curves of resonant modes. (**a**) Simulated spectra of local E-field enhancement for patch nanoantennas with patch diameters of 104 nm (blue) and 142 nm (red) respectively; (**b**) Calculated and (**c**) measured reflectance spectra of patch nanoantennas arrays with 104 nm and 142 nm patches diameters; (**d**) Calculated dispersion curves of patch nanoantennas with gap material being air (blue) and SiO_2_ (red). Cyan circles represent the fundamental modes resonating at 765/645 nm wavelength, while green triangles represent the second order modes resonating at 767/645 nm wavelength. All the calculations and measurements are for the normal incidence.

To further understand the mechanism responsible for this enhancement, we performed numerical simulations using CST Microwave Studio. In the simulations, we fixed the array periodicity at 400 nm and the SiO_2_ thickness at 30 nm. The patches diameters were set to be 104 nm and 142 nm. Complex dielectric constants of Au were obtained by fitting the data from Johnson and Christy^28^. E-field probes were placed at the edge of the patches to obtain the local field enhancement spectra (Fig. 4a). A set of field-monitor-planes placed at 800 nm above the patches were used to calculate the reflection spectra (Fig. 4b). Under the normal incidence of white light, the spectra of local field enhancement clearly show a resonance peak around 650 nm for 104-nm antennas. When increasing the diameter to 142 nm, the local field enhancement peak is shifted to 770 nm (Fig. 4a). Correspondingly, the calculated reflection spectra show large dips at 650 nm for 104-nm antennas and 770 nm for 142-nm antennas (Fig. 4b), which well matches the 645 nm and 767 nm of our experimental measurements (Fig. 4c).

Previous studies have shown that the resonant conditions of circular patch nanoantennas can be approximately expressed in the following:


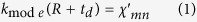


where *k*_*mode*_is the wave number of the resonant modes. *R* and *t*_*d*_ represent the patch radius and the gap thickness respectively. 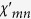
denotes the *n*-th root of the first derivative of the Bessel function of the *m*-th kind. The fundamental mode, i.e., the first order mode, happens when both m and n are equal to 1, while the higher order modes require m and/or n to be larger than 1^29^,^30^. Assuming that the patches of the antennas have a finite thickness *t*_m_, the dispersion for the relevant anti-symmetric modes satisfy the following (see Supporting Information S5 for derivation):


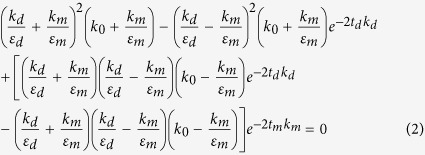


where 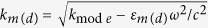, *k*_*d*_ and *k*_*m*_ are the wave number in dielectric and metal patches respectively. *ε*_*d*_ and *ε*_*m*_ represent the complex dielectric constants of the gap material and the metal patches respectively.

Based on Equation 1, we can calculate the resonant wave numbers of the fundamental modes and the other high order modes. Based on Equation 2, we can plot whole dispersion curves (i.e. resonant wavelengths as the function of wave numbers). As shown in Fig. 4d, the blue and red dispersion curves correspond to the dielectric materials being totally in air and SiO_2_ respectively. By comparing the resonant wave numbers obtained from Equations 1 and 2, we found that the resonant wavelengths of 645 nm and 767 nm could only be for the first order modes (the cyan circles in Fig. 4d), which usually provides the strongest local field enhancements^26^. It should be mentioned that in this study the simulated or measured resonant wave numbers do not exactly match up with the SiO_2_ dispersion curve, but are located within the enclosed region by the air and SiO_2_ dispersion curves as shown in Fig. 2d. The reason for this mismatch lies in the fact that the dielectric layer is not pure SiO_2_ but, rather, air outside the patches. This mismatch implies that the fringe field in the air is very strong and can be utilized for g-NQDs emission enhancement.

**Figure 5:**
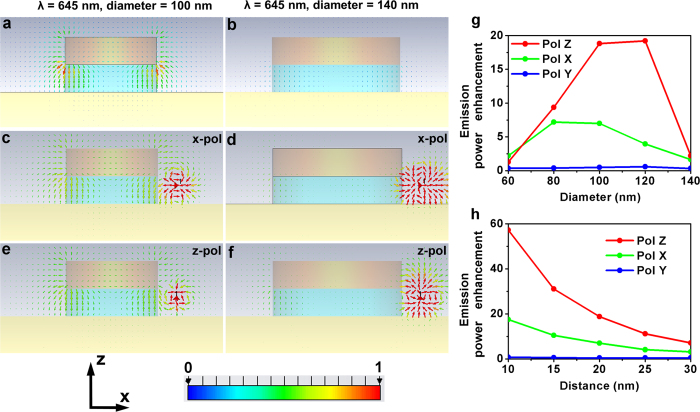
Near-field distribution at 645 nm for 104-nm (**a**,c,e) and 142-nm (**b**,**d**,**f**) patch nanoantennas. (**a**) and (**b**) are excited by normal incident light; (**c**,**d**) are excited by x-polarized dipoles at the fringe of the antennas; (**e**,**f**) are excited by z-polarized dipoles at the fringe of the antennas; (**g**) Emission enhancement at 645 nm as a function of the patch diameters with the distance between dipoles and patch edges fixed at 20 nm; (**h**) Emission power enhancement at 645 nm as a function of the distances between the dipoles and the patch edges for a 104 nm diameter patch antenna.

For 104-nm antennas excited normally by white light, the simulated near-field distribution further confirms that the fringe field is very strong at the mode wavelength, i.e., 645 nm (Fig. 5a). Conversely, x-polarized or z-polarized dipoles placed in the fringe area of the 104-nm antennas can strongly couple with the antenna resonances and then transmit to the far-field region (Fig. 5c,e). As a comparison, the fringe field around the 142-nm antennas is negligible since there exists no resonant modes at 645 nm (Fig. 5b). Consequently, fringe dipoles cannot effectively interact with the antennas (Fig. 5d,f). In these simulations, we assumed that the dipole is located at the center of the QDs. Due to the thick silica shell that surrounds the QD, the center of the coupled QD is about 20 nm above the bottom Au film and ~20 nm away from the edges of the patches as in Fig. 5c–f.

Next to determine emission power enhancement, we first calculate the emitted power of the dipoles with and without coupling to the patch nanoantennas in +z direction by integrating the Poynting vectors transmitting through the field monitor plane which is placed 800 nm above the antennas. The emission power enhancement is then obtained as the ratio between the two integrated Poynting vectors. Figure 5g displays the emission power enhancement at 645 nm as the function of patch diameter. Clearly, for patches diameters within the range of 80–120 nm, strong enhancements can be expected, while for diameters out of this range, enhancements decrease rapidly. In addition, the emission enhancement is also highly sensitive to the distances between the g-NQDs and the patch edges. For 104 nm patches, the enhancement values exponentially decrease with increasing the QD-patch edge distances as shown in Fig. 5h. From Fig. 5g,h, we can also see that, while the y-polarized dipolar emitters can hardly couple to the patch nanoantennas, both of the emissions from x- and z-polarized emitters can receive strong enhancements. Interestingly, the enhancement on z polarized emitters is shown to be ~4 times stronger (5 vs 20, Fig. 5g) than the maximum enhancement possible in case of the x polarized dipole. This ability to enhance z-polarized dipoles, we believe, uniquely distinguishes patch antenna over other types of antennas such as parallel gap-bars^17^,^24^.

**Figure 6:**
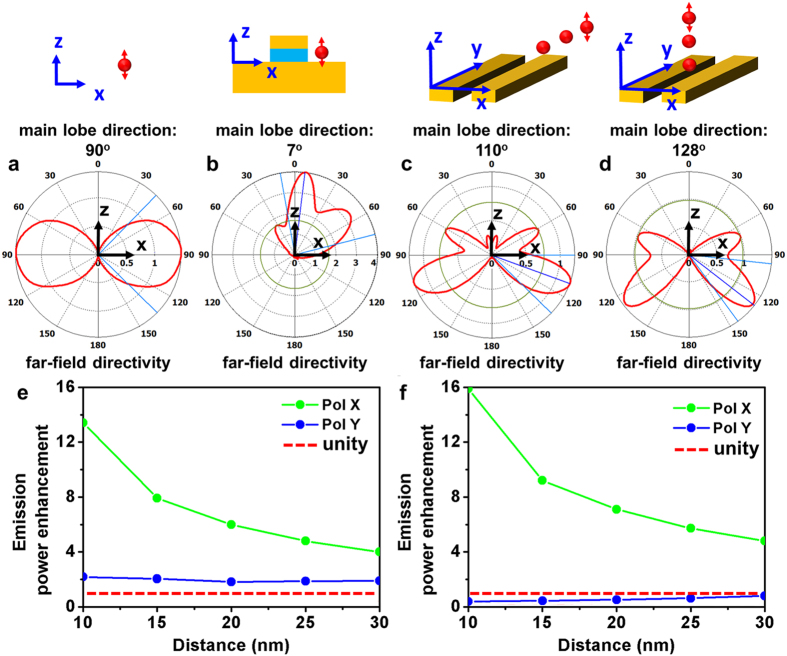
Radiation directivity (in x-z plane) for a z-polarized dipole (**a**) in free space; (**b**) coupled to 104 nm patch nanoantenna through fringe field (20 nm separation); (**c**) in-plane coupled to one end of the gap-bar antenna (20 nm separation along y direction); (**d**) above-plane coupled to the gap-bar antenna (20 nm separation along z direction). Emission power enhancement at 645 nm wavelength as a function of the separation distances between the polarized dipoles and the gap-bar antennas. (**e**) separation along y direction; (**f**) separation along z direction. Red-dotted lines in Fig. 6e,f represent the unity enhancement.

Because the main lobes of radiation for an uncoupled z-polarized dipole lie in x-y plane as shown in Fig. 6a, little or no emission is coupled into the 0.85 NA collection objective located directly above the sample. However when a z-polarized dipole is coupled to the fundamental mode of patch antenna, a strong x-polarized dipole is induced in the patch antenna yielding emission directed along the z axis. In addition, the original dipole is partially cancelled by a z-polarized resonating electric field with opposite phase, which is induced at the opposite edge of the antenna. The emission in x-y plane, as a result, is strongly suppressed and the main emission lobe is oriented 7° to the z-axis emerges as demonstrated in Fig. 6b. As majority of this emission can be coupled to the collection objective, the strong enhancement in emission is attained as the combined result of redirection of radiation and enhancement of dipole. While the fields extending out of the walls of the gap-bar antenna in both z and y direction (Fig. 6c,d upper panels) can be considered as the fringe fields, our calculation for radiation lobes of the z polarized dipole coupled to these in-plane (Fig. 6c, lower panel) and above-plane fringe fields (Fig. 6d, lower panel) show that the redirection of radiation lobe toward z-direction cannot occur for both configurations.

Furthermore calculations for the power enhancements for these in-plane and above-plane coupling configurations revealed that the strongest enhancement of the x-polarized dipole (~8 at 20 nm for both Fig. 6e,f) is slightly smaller than that demonstrated in case of patch antenna (~20 Fig. 5g). For this calculation we set the gap of the bars to be 40 nm which is the minimum value allowed by the diameter of the silica coated g-NQDs. In case of y polarized dipole, while a slight enhancement ~2 is observed for in-plane coupling (Fig. 6c,e), a suppression is resulted in above-plane coupling (Fig. 6d,f). Over all, these calculations show that the gap-bar antennas, in comparison to the patch antennas, provide a smaller enhancement, further confirming the advantages of fringe field couplings in patch nanoantennas. Here we also note that, according to the simulation (see Supporting Information S3 and S4), the excitation enhancements for both 104- and 142-nm antennas are negligible since they do not support any resonant modes at 405 nm wavelength of the lasers.

In conclusion, we have fabricated patch nanoantennas with their fundamental mode in resonance with the emission wavelength of CdSe/CdS/SiO_2_ core/shell/shell g-NQDs. Simulations and theoretical analyses show that the fundamental mode has a very strong fringe field so that g-NQDs positioned around the patches could strongly couple to the antennas. Under CW laser excitation, the antennas in resonance with the fringe-region g-NQDs show a much larger radiative enhancement than that of the antennas out of resonance. In addition, we have made a comparison between the fringe coupled g-NQDs and the g-NQDs well-aligned in gap-bar nanoantennas, where they show similar enhancement capabilities. These experiments proved the effectiveness of fringe coupling methods. We have also demonstrated that, without enhancing the laser excitation and quenching the single exciton emission, the bi-exciton quantum yield of single g-NQDs can be effectively enhanced by fringe coupling to the patch nanoantennas.

## Additional Information

**How to cite this article**: Wang, F. *et al.* Coupling Single Giant Nanocrystal Quantum Dots to Fundamental Modes of Patch Nanoantennas through Fringe Field. *Sci. Rep.* **5**, 14313; doi: 10.1038/srep14313 (2015).

## Supplementary Material

Supplementary Information
