# Supplementary material for: Coupling Single Giant Nanocrystal Quantum Dots to the Fundamental Mode of Patch Nanoantennas through Fringe Field
Source: Sci Rep. 2015 Sep 23;5:14313. doi: 10.1038/srep14313 (PMC4585802; doi:10.1038/srep14313)
Supplement: Supplementary Information [file srep14313-s1.pdf]

Supporting Information

Coupling Single Giant Nanocrystal Quantum Dots to Fundamental Modes of Patch Nanoantennas through Fringe Field

Feng Wang, Niladri S. Karan, Hue Minh Nguyen, Yagnaseni Ghosh, Jennifer A. Hollingsworth and Han Htoon*

Center for Integrated Nanotechnologies, Materials Physics & Applications Division, Los Alamos National Laboratory, Los Alamos, New Mexico 87545
*htoon@lanl.gov

S1. Synthesis of the studied CdSe/CdS/SiO_2 g-NQDs and lifetime and g2 statistics for the g-NQDs with and without coupling to 104 nm patch nanoantennas
S2. A representative SEM image of the fabricated g-NQD-gap bar antenna integrated structure and the simulated emission enhancement spectra of the antenna.
S3. Simulated local field enhancement around the 405-nm laser excitation wavelength.
S4. Poissonian distribution model for the pump-power dependent PL intensity.
S5. Derivation of the dispersion curve of patch nanoantennas.

S1. Synthesis the studied CdSe/CdS/SiO_2 g-NQDs and lifetime and g2 statistics for the g-NQDs with and without coupling to 104 nm patch nanoantennas.

The standard Successive Ionic Layer Adsorption and Reaction (SILAR) approach with a higher shell-growth temperature was adopted for synthesizing CdSe/CdS core, with a 4-nm-thick shell (16 monolayer) g-NQDs.[1,2] A reverse microemulsion method was used to perform the silica coating of the hydrophobic g-NQDs.[3]

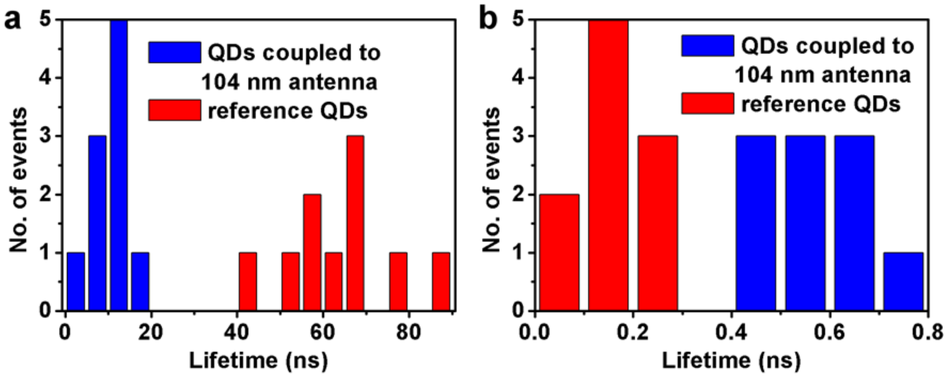

Figure S1) | a) The statistics of lifetimes for reference g-NQDs and g-NQDs coupled to 104 nm patch nanoantennas; b) The statistics of g2 values for reference g-NQDs and g-NQDs coupled to 104 nm patch nanoantennas.

S2. A representative SEM image of the fabricated g-NQD-gap bar antenna integrated structure and the simulated emission enhancement spectra of the antenna.

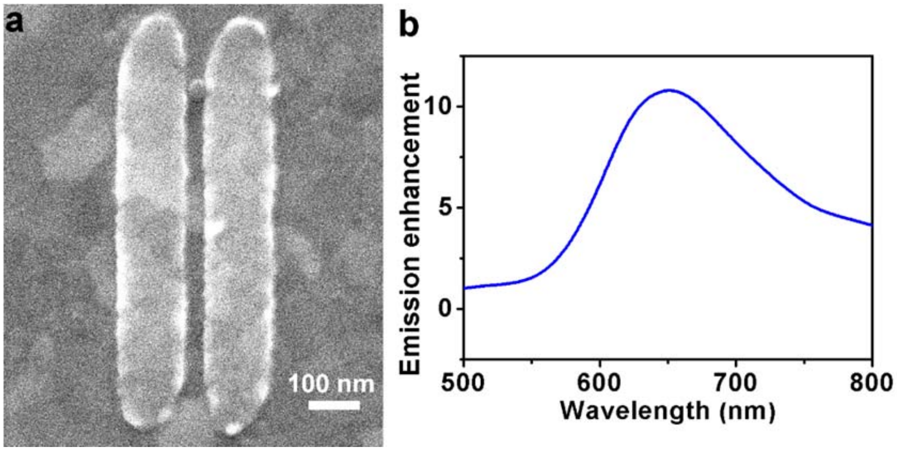

Figure S2 | (a) SEM image of the fabricated single g-NQD-gap bar nanoantenna coupled structure utilizing two-step e-beam lithography; (b) The simulated emission enhancement spectrum for this g-NQD positioned inside a 50 nm antenna gap. The peak wavelength of the enhancement spectrum is 650 nm, which coincides with the emission band of the $CdSe/CdS/SiO_2$ g-NQD.

The fabricated gap bar nanoantennas have a 140-nm bar width, 900-nm bar length, and 45-nm thickness. The gap between the two bars is approximately 50 nm. The same geometric parameters are used to perform the simulation. The calculated local field enhancement is for light normal incident on the patch nanoantennas. In the simulation, an E-field probe was placed 20 nm away from the patches and 20 nm above the Au ground since the radius of the g-NQDs is about 20 nm.

S3. Simulated local field enhancement around the 405-nm laser excitation wavelength.

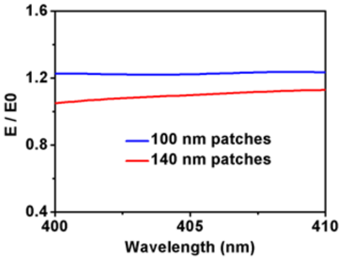

Figure S3 | The simulated local field enhancement for patch nanoantennas with 100-nm patches (blue) and 142-nm patches (red) with ~405-nm wavelength (the laser excitation wavelength).

S4. Poissonian distribution model for the pump-power dependent PL intensity.

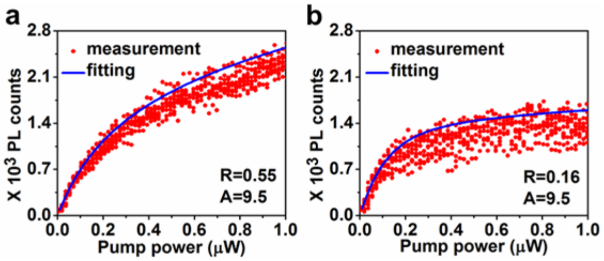

Figure S4 | (a) and (b) measured (red) and fitted (blue) PL intensity of single g-NQDs as the function of laser pump power. While (a) corresponds to one single g-NQD placed on the 104 nm patch nanoantenna, (b) corresponds to one single g-NQD placed on glass for reference. The fit to the measured saturation was performed for the data points in the top 5% of intensity since those data in all likelihood are the emissions from neutral excitons having near-unity quantum yield.

Upon photoexcitation with a laser pulse, one NQD can absorb N photons and form an N-excitons state with N obeying the Poisson distribution, i.e.,

$$P(N, \langle N \rangle) = \langle N \rangle^{N} e^{-\langle N \rangle} / N!. \tag{S1}$$

Here, $\langle N \rangle$ is the average NQD occupancy per excitation pulse, depending on the absorption cross-section of g-NQDs (σ), laser power w, laser repetition rate R, excitation spot area D and photon energy E_{ph}.

$$\langle N \rangle = Aw, \text{ with } A = \sigma / RDE_{ph}. \tag{S2}$$

The PL intensity from this NQD can then be modeled as

$$I(w) = C \sum_{N=1}^{\infty} P(N, \langle N \rangle) \sum_{m=1}^{N} Q_{mx}. \tag{S3}$$

Here, C is a constant mainly representing the photon collection efficiency of the measurement system. Q_{mX} is the quantum yield of m-exciton state and can be calculated from Q_{2X}:

$$Q_{mx} = \frac{1}{1 + (m-1)(1 - Q_{2x}) / Q_{2x}} \tag{S4}$$

Equations S1, S2, S3, and S4 together allow us to model the PL saturation behavior of single g-NQDs in terms of the bi-exciton quantum yields[4] and the proportionality constant A that provide a direct measure of σ, absorption cross-section.

The PL intensity (red circles) as the function of laser pump power are shown in Figure S4 (a) and (b), with (a) representing one g-NQD placed on the 104-nm patch nanoantennas and (b) representing a reference g-NQD placed on glass substrate. While the reference g-NQD shows an apparent emission saturation behavior, the emission of g-NQDs on antennas maintains a rapid increase with the increasing pump power due to its large bi-exciton quantum yield. The fitting to the pump dependent PL (blue curves) in Figure S4 (a) and (b) gives exactly the same value of A (i.e., 9.5), which indicates that g-NQDs on glass and on antennas have the same absorption cross sections and thus the same local excitation power. Consequently, the enhanced radiative decay rate and bi-excitation quantum yield observed for g-NQDs-antenna-coupled structures should be attributable to the emission-band coupling instead of the excitation enhancement.

S5. Derivation of the dispersion curve of patch nanoantennas.

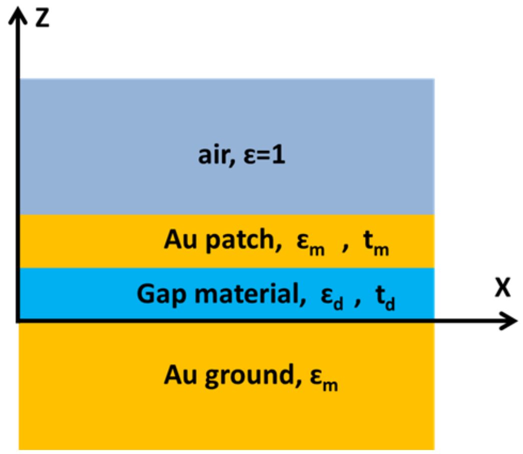

Figure S5 | Schematic of the studied air-Au patch-dielectric space-Au ground structure.

The surface plasmons can only be excited by a TM wave so we can set the direction of the magnetic field as the y-direction. Then, only H_y, E_x, and E_z components exist in the studied structure, and they satisfy the following:

$$\frac{\partial^2 H_y}{\partial z^2} + \left(k_0^2 \varepsilon_0 \varepsilon_j - \beta^2\right) H_y = 0$$

$$E_x = -i \frac{1}{\omega \varepsilon_0 \varepsilon_j} \frac{\partial H_y}{\partial z} \tag{S5}$$

$$E_z = -\frac{\beta}{\omega \varepsilon_0 \varepsilon_j} H_y$$

ε_j is the complex dielectric constant of materials. H_y takes the form:

$$H_y = H_y(z) e^{i\beta x} e^{-i\omega t}$$

Here, the phase factor $e^{i\beta x} e^{-i\omega t}$ can be ignored in the analysis. Considering the continuation of the tangential component of electric and magnetic fields, we only need to discuss $H_y(z)$ and $E_x(z)$.

For $z \leq 0$,

$$H_y(z) = A e^{k_m z}$$

$$E_x(z) = -iAk_m \frac{1}{\omega \varepsilon_0 \varepsilon_m} e^{k_m z} \tag{S6}$$

For $0 < z \leq t_d$, the surface plasmons are coupled,

$$H_y(z) = B e^{k_d z} + C e^{-k_d z}$$

$$E_x(z) = -iBk_d \frac{1}{\omega \varepsilon_0 \varepsilon_d} e^{k_d z} + iCk_d \frac{1}{\omega \varepsilon_0 \varepsilon_d} e^{-k_d z} \tag{S7}$$

For $t_d < z \leq t_d + t_m$,

$$H_y(z) = D e^{k_m z} + E e^{-k_m z}$$

$$E_x(z) = -iDk_m \frac{1}{\omega \varepsilon_0 \varepsilon_m} e^{k_m z} + iEk_m \frac{1}{\omega \varepsilon_0 \varepsilon_m} e^{-k_m z} \tag{S8}$$

For $z > t_d + t_m$,

$$H_y(z) = F e^{-k_0 z}$$

$$E_x(z) = iFk_0 \frac{1}{\omega \varepsilon_0} e^{-k_0 z} \tag{S9}$$

At z=0, we have the boundary condition:

$$A=B+C$$

$$-A \frac{k_m}{\varepsilon_m} = -B \frac{k_d}{\varepsilon_d} + C \frac{k_d}{\varepsilon_d} \tag{S10}$$

at $z = t_d$,

$$\mathrm{D} e^{k_m t_d} + E e^{-k_m t_d} = B e^{k_d t_d} + C e^{-k_d t_d}$$

$$-Bk_d \frac{1}{\varepsilon_d} e^{k_d t_d} + Ck_d \frac{1}{\varepsilon_d} e^{-k_d t_d} = -Dk_m \frac{1}{\varepsilon_m} e^{k_m t_d} + Ek_m \frac{1}{\varepsilon_m} e^{-k_m t_d} \tag{S11}$$

at $z = t_d + t_m$,

$$\mathrm{D} e^{k_m (t_d + t_m)} + E e^{-k_m (t_d + t_m)} = F e^{-k_0 (t_d + t_m)}$$

$$-Dk_m \frac{1}{\varepsilon_m} e^{k_m (t_d + t_m)} + Ek_m \frac{1}{\varepsilon_m} e^{-k_m (t_d + t_m)} = Fk_0 e^{-k_0 (t_d + t_m)} \tag{S12}$$

By solving boundary condition equations, i.e. S10, S11 and S12, we can obtain the dispersion relation as mentioned in the main manuscript:

$$
\left(\frac{k_d}{\varepsilon_d}+\frac{k_m}{\varepsilon_m}\right)^2\left(k_0+\frac{k_m}{\varepsilon_m}\right)-\left(\frac{k_d}{\varepsilon_d}-\frac{k_m}{\varepsilon_m}\right)^2\left(k_0+\frac{k_m}{\varepsilon_m}\right)e^{-2t_dk_d}
$$

$$
+\left[\left(\frac{k_d}{\varepsilon_d}+\frac{k_m}{\varepsilon_m}\right)\left(\frac{k_d}{\varepsilon_d}-\frac{k_m}{\varepsilon_m}\right)\left(k_0-\frac{k_m}{\varepsilon_m}\right)e^{-2t_dk_d}-\left(\frac{k_d}{\varepsilon_d}+\frac{k_m}{\varepsilon_m}\right)\left(\frac{k_d}{\varepsilon_d}-\frac{k_m}{\varepsilon_m}\right)\left(k_0-\frac{k_m}{\varepsilon_m}\right)\right]e^{-2t_mk_m}=0 \qquad (S13)
$$

References

1. Ghosh, Y. et al. New insights into the complexities of shell growth and the strong influence of particle volume in nonblinking "giant" core/shell nanocrystal quantum dots. *J. Am. Chem. Soc.* **134,** 9634–9643 (2012).
2. Chen, Y. et al. "Giant" multishell CdSe nanocrystal quantum dots with suppressed blinking. *J. Am. Chem. Soc.* **130,** 5026 (2008).
3. Yi, D. K. et al. Silica-coated nanocomposites of magnetic nanoparticles and quantum dots. *J. Am. Chem. Soc.* **127,** 4990 (2005).
4. Park, Y.-S. et al. Super-Poissonian Statistics of Photon Emission from Single CdSe-CdS Core-Shell Nanocrystals Coupled to Metal Nanostructures. *Phys. Rev. Lett.* **110**,117401 (2013).
